# Treatment Modalities and Antimicrobial Stewardship Initiatives in the Management of Intra-Abdominal Infections

**DOI:** 10.3390/antibiotics5010011

**Published:** 2016-02-15

**Authors:** Charles Hoffmann, Matthew Zak, Lisa Avery, Jack Brown

**Affiliations:** 1 Wegmans School of Pharmacy, St. John Fisher College, Rochester, NY 14618, USA; choffmann@sjfc.edu (C.H.); mzak@sjfc.edu (M.Z.); lavery@sjfc.edu (L.A.); 2 Department of Pharmacy, University of Rochester Medical Center, Rochester, NY 14642, USA; 3 St. Joseph’s Hospital Health Center, Syracuse, NY 13203, USA; 4 Department of Medicine, University of Rochester Medical Center, Rochester, NY 14642, USA

**Keywords:** antimicrobial stewardship, intra-abdominal infection, appendicitis, cholecystitis, diverticulitis

## Abstract

Antimicrobial stewardship programs (ASPs) focus on improving the utilization of broad spectrum antibiotics to decrease the incidence of multidrug-resistant Gram positive and Gram negative pathogens. Hospital admission for both medical and surgical intra-abdominal infections (IAIs) commonly results in the empiric use of broad spectrum antibiotics such as fluoroquinolones, beta-lactam beta-lactamase inhibitors, and carbapenems that can select for resistant organisms. This review will discuss the management of uncomplicated and complicated IAIs as well as highlight stewardship initiatives focusing on the proper use of broad spectrum antibiotics.

## 1. Introduction

Intra-abdominal infections (IAIs) are the second most common cause of infection-related mortality in intensive care units [1]. Complicated IAIs extend into the peritoneal space and are associated with abscess formation or peritonitis. Common IAIs include appendicitis, diverticulitis and cholecystitis [1,2]. Recommendations for the diagnosis and management of complicated IAIs are reviewed in the Surgical Infection Society and the Infectious Diseases Society of America (SIS-IDSA) Guideline in 2010, the World Society of Emergency Surgery (WSES) Expert Panel in 2013 and the Tokyo Guidelines (TG13) for acute cholangitis and acute cholecystitis in 2013 [2,3,4]. These guidelines recommend antimicrobial therapy based on the severity and etiology of infection as well as the presence of underlying medical conditions. Antimicrobials used for empiric therapy of IAIs should be active against enteric Gram negative aerobic and facultative anaerobic bacilli and enteric Gram positive streptococci. Coverage for obligate anaerobic bacilli should be added for infections of the distal small bowel, appendix or colon; given the complexity of IAIs, anti-anaerobic coverage is routinely utilized. The treatment of IAIs involves a balance between optimizing antimicrobial therapy and reducing collateral damage of antimicrobial use, including emerging resistance and *Clostridium difficile* infections (CDI) [5,6]. The purpose of this review is to discuss the contemporary management of complicated IAIs as well as identify areas for antimicrobial stewardship.

## 2. Appendicitis

### 2.1. Epidemiology/Pathogenesis

The appendix is the most common source of infection in community-acquired IAIs [7]. The incidence of appendicitis increases throughout childhood and peaks at 15–25 years of age; roughly 300,000 appendectomies are performed annually in the United States [8,9]. The primary pathogenic mechanism of acute appendicitis is obstruction of the appendiceal lumen, which can occur secondary to mucus plugging, congregated stool or foreign body [8,10,11]. Appendiceal lumen obstruction leads to increased intraluminal pressure and subsequent compression of lymphatic and vascular drainage causing ischemic damage of the mucosa. Microbial invasion also occurs during this process. If left unaddressed, continued inflammation and ischemia may lead to gangrene and perforation [8].

### 2.2. Microbiology

Appendiceal infections are often polymicrobial; complicated IAIs secondary to appendicitis commonly involve colonic microflora [8]. The Study for Monitoring Antimicrobial Resistance Trends (SMART) has monitored antimicrobial susceptibility data for IAIs globally since 2002 [12]. Lob and colleagues analyzed SMART data from 2008 to 2010 and reviewed the occurrence and susceptibility of organisms in appendicitis-associated IAIs. Overall, the most common isolates identified in adult patients were *Escherichia coli* (*E. coli*) (68.3%), with *Klebsiella pneumoniae* (*K. pneumoniae*) (10.1%) and *Pseudomonas aeruginosa* (*P. aeruginosa*) (6.9%) ranking a distant second or third depending on geographic location [12]. Additionally, authors found a lower rate of extended-spectrum beta-lactamase (ESBL) producing *E. coli*, *K. pneumoniae*, *Klebsiella oxytoca*, and *Proteus mirabilis* among patients with appendicitis-associated IAIs compared with non-appendicitis-associated IAIs [12]. Authors commented that this finding may be due to the fact that appendicitis-associated pathogens generally arise from normal bowel flora and do not reflect the higher ESBL rates found in health care-associated IAIs [12]. Of importance, routine use of intra-operative culture swabs is not recommended by several stakeholders, as studies found that changes in antimicrobial regimens based on intra-operative culture results usually made little difference in clinical outcomes [7,12,13]. Therefore, it is important for stewardship teams to be familiar with the general epidemiology of appendicitis-related infections, as well as current regional and local susceptibility patterns to guide empiric antibiotic therapy in patients with appendicitis.

### 2.3. Clinical Presentation/Diagnosis

Appendicitis starts as colicky, visceral periumbilical pain that evolves over the next 6–24 h to localized, somatic right lower quadrant abdominal pain [8]. Pain is often accompanied by mild fever, nausea, vomiting and anorexia. Imaging studies aid in diagnosing as ultrasonography is rapid, noninvasive and particularly useful for pregnant women and children. Additionally, computed tomography (CT), given reported sensitivity and specificity rates of 94% and 95%, respectively, has been shown to help diagnose other pathologic conditions and prevent unnecessary hospitalizations [14,15,16]. Despite the fact that ultrasonography and CT have become more readily available, clinical examination remains the cornerstone of the diagnostic process and should not be neglected [17].

### 2.4. Treatment

The definitive treatment for appendicitis is surgery which includes preoperative fluid resuscitation and prophylactic parenteral antibiotics [8]. An appendectomy is a successful treatment procedure with good results; however, complications, most notably wound infections, intra-abdominal abscess formation and ileus, are inherent to operative therapies [18,19]. A contemporary strategy in the management of appendicitis involves treatment with antibiotics and performing an appendectomy only if the appendicitis does not resolve or reoccurs; this is known as an “antibiotics first” strategy [20]. Several randomized trials comparing an antibiotics first strategy to appendectomy upon presentation for uncomplicated appendicitis show varying rates of appendectomies in the antibiotics first group [21,22].

A literature search was conducted using PubMed and the search terms “appendectomy”, “antibiotics” and “randomized control trial”. The search was limited to adults and studies from 2010 to present. Vons and colleagues conducted a multicenter, open-label, non-inferiority, randomized controlled trial comparing results of treatment with amoxicillin-clavulanic acid with emergency appendectomy in patient with uncomplicated acute appendicitis [21]. Patients received amoxicillin-clavulanic acid because of its efficacy in treating uncomplicated sigmoiditis [23]. Antibiotic therapy was given intravenously to patients with nausea or vomiting, and orally to all others. Upon discharge, patients continued amoxicillin-clavulanic acid at the same dose for eight days and were seen on Day 8; if patients had a sustained high white blood cell (WBC) count or elevated C-reactive protein (CRP), antibiotics were continued for another eight days with surgical intervention on Day 15 if laboratory abnormalities persisted. The primary endpoint was occurrence of peritonitis within 30 days of initial treatment and authors used a non-inferior margin of 10%. Diagnosis of peritonitis amongst patients randomized to receive antibiotic therapy occurred when a complicated appendicitis was identified via appendectomy. Patients randomized to antibiotic therapy had significantly higher rates of 30 day post-intervention peritonitis compared with the appendectomy group (8% *vs.* 2%, treatment difference 5.8; 95% CI, 0.3–12.1). Of the 120 patients randomized to antibiotic therapy, 39 underwent appendectomy due to acute appendicitis within one-year follow up; of the 14 procedures that occurred within 30 days, nine were due to complicated appendicitis. Authors concluded that antibiotic therapy with amoxicillin-clavulanic acid was not non-inferior to emergency appendectomy for the treatment of acute uncomplicated appendicitis. Authors cited limitations with the study and commented that patients with complicated appendicitis may have mistakenly been included and randomized. Additionally, evidence shows increasing resistance of *E. coli* to amoxicillin-clavulanic acid, thus possibly affecting treatment outcomes [21].

Salminen and colleagues conducted a multicenter, open-label, non-inferiority, randomized controlled trial comparing antibiotic treatment with appendectomy for the treatment of uncomplicated acute appendicitis [22]. The primary end point for patients in the antibiotic group was resolution of acute appendicitis, resulting in hospital discharge without surgical intervention and no recurrent appendicitis during a minimum follow up period of one year. Patients randomized to antibiotic treatment received intravenous ertapenem 1 g daily for three days followed by seven days of oral levofloxacin (500 mg once daily) and metronidazole (500 mg three times per day). Of the 1379 patients assessed for eligibility, 273 were randomized to receive an appendectomy and 257 received a nonsurgical approach with antibiotic therapy. One patient in the antibiotic group was lost to follow up, so of the 256 patients randomized to antibiotic therapy and followed, 70 (27.3%; 95% CI, 22.0%–33.2%) required an appendectomy within one year of initial presentation. The intent-to-treat analysis generated a difference amongst treatment groups of −27.0% (95% CI, −31.6% to ∞; *p* = 0.89). Because of the pre-defined 24% minimal clinically important difference, authors did not demonstrate non-inferiority of antibiotic therapy for appendicitis compared to appendectomy. However, patients who were initially treated with antibiotics but underwent appendectomy within one year due to recurrent appendicitis had an overall lower surgical complication rate than those patients originally randomized to receive an appendectomy (7.0% *vs.* 20.5%, respectively). Additionally, the majority of patients (58 of 70) who initially received antibiotics yet went on to have an appendectomy had surgical intervention due to uncomplicated appendicitis which challenges the notion that lack of prompt surgical intervention leads to perforation. Limitations of this trial include the sample size; appendectomy is considered the treatment of choice for acute appendicitis, thus researchers had difficulty enrolling patients in the study and therefore had to recalculate the sample size which affected power. Of note, this study excluded pregnant women and patients with complicated appendicitis, so the results do not apply to these groups [22]. Several meta-analysis and systematic reviews have compared antibiotic therapy with appendectomies in the treatment of acute appendicitis [24,25,26,27,28]. These reviews demonstrate higher success rates with surgery but also higher rates of complications. Limitations of these reviews include selection bias, high crossover rates and inclusion of randomized-controlled trials of poor methodological quality, thus overall conclusions differed [24,25,26,27,28].

### 2.5. Conclusion

In the trials discussed above, antibiotic therapy for acute appendicitis did not meet non-inferiority when compared with appendectomy; however, study limitations affect definitive conclusions. Risks are inherent to surgical procedures and must be weighed against the risk of recurrent appendicitis with an antibiotics first strategy.

## 3. Diverticulitis

### 3.1. Epidemiology/Pathogenesis

Diverticulosis describes the presence of diverticula, which is an exposure of mucosa through the colonic wall. Diverticulitis describes the inflammation of these diverticula, causing troublesome symptoms such as abdominal pain, fever, tenderness, constipation and trace blood from the rectum [29,30]. Diverticular disease becomes more common with advanced age; it is estimated that approximately 70% of adults will have some level of diverticular disease by age 80, although only approximately 10%–25% of these cases become clinically significant [29,30,31]. The most understood global risk factor for developing diverticulosis and diverticulitis at any point in life is low intake of dietary fiber, though the use of systemic corticosteroids, narcotics and non-steroidal anti-inflammatory drugs (NSAIDs) may also play a role [30].

### 3.2. Microbiology

Disorders of the gastrointestinal tract, including diverticular disease, are postulated to involve disturbances of the normal balance of colonic bacteria [32]. Bacterial overgrowth leading to alterations in intestinal flora can also induce low-grade inflammation of the mucosa. Similar to other IAIs, diverticulitis is a polymicrobial infection caused by endogenous facultative bacteria and anaerobes [30,33]. Brook and colleagues analyzed the microbiology of IAIs associated with diverticulitis and found that the most common organisms isolated from peritoneal fluid and intra-abdominal abscesses were *E. coli* (65.5% and 68.2%, respectively), *Bacteroides fragilis* (40% and 63.6%, respectively), gamma-hemolytic streptococci (12.7% and 9.1%, respectively) and alpha-hemolytic streptococci (10.9% and 27.3%, respectively) [32,33].

### 3.3. Clinical Presentation/Diagnosis

Diagnosis of diverticulitis is made through the combination of a physical examination as well as lab and imaging results. Though a physical exam may be unremarkable, it most likely will reveal left-lower quadrant pain, abdominal tenderness and potentially an abdominal mass. Fever and leukocytosis may also be present, but those findings are non-specific [30]. Uncomplicated diverticulitis is described as localized inflammation that may or may not have small abscess formation within the bowel wall only. Diverticulitis can be considered complicated if an abscess is present anywhere besides the bowel wall with or without the presence of fistula, stricture, peritonitis or sepsis [34]. Tests done to diagnose diverticulitis include a barium enema, colonoscopy and CT imaging, though the most common diagnostic imaging utilized is CT, which shows approximately 84% sensitivity and 87% specificity, respectively [29]. The disease can also be given a Modified Hinchey Classification, which is a system used to categorize progression of disease based on radiographic findings. Originally developed by Hinchey and colleagues in 1978 and modified by Wasvary and colleagues in 1999, the ranking system has been widely adopted and utilizes a six point scale classifying complicated diverticulitis from stage 0 (mild clinical diverticulitis) to stage IV (generalized fecal peritonitis) [35,36].

### 3.4. Treatment

Several factors influence antimicrobial treatment of diverticulitis, including the severity of illness upon presentation as well as the presence of risk factors for poor clinical outcomes [37]. A literature search was conducted using PubMed and the search terms “diverticulitis treatment”, “diverticular disease”, “diverticulitis guidelines” and “diverticulitis antibiotics”. The search was limited to adults and studies from 2010 to present. Several recent publications have questioned the use of antibiotics in uncomplicated diverticulitis. Shabanzadeh and Wille-Jorgenson performed a review of randomized controlled trials analyzing the use of antibiotics to treat uncomplicated diverticulitis [38]. Three randomized controlled trials were analyzed and will be discussed herein. Chabok and colleagues conducted a multicenter, randomized trial comparing outcomes in patients with CT-verified acute uncomplicated left-sided diverticulitis randomized to treatment with or without antibiotics [39]. Overall, 623 patients were enrolled in the study; 314 patients received antibiotics while 309 did not, respectively. The primary endpoint was occurrence of complications, such as the formation of an abscess, free air or fistula, at 12 month follow up interval. There was no significant difference between groups regarding occurrence of complications as 3/314 patients who received antibiotics and 6/309 patients who did not receive antibiotics suffered complications, respectively [39]. Ribas and colleagues conducted a randomized trial to compare outcomes in patients with CT-confirmed uncomplicated diverticulitis who received oral antibiotics after a short course of intravenous antibiotic therapy *vs.* patients who received longer courses of intravenous antibiotics [40]. All patients received intravenous amoxicillin-clavulanic acid 1 g every 8 h. After 24–48 h and when able to tolerate a liquid diet, patients randomized to the short course of antibiotic therapy were transitioned to oral amoxicillin-clavulanic acid at the same dose and continued for 10 additional days. Meanwhile, patients randomized to a longer course of intravenous antibiotic therapy were transitioned to oral amoxicillin-clavulanic acid after Day 7 and continued on oral therapy for five additional days. Treatment failure was defined as the inability to discharge the patient secondary to persistent pain and vomiting, emergency admission secondary to symptoms of previous diverticulitis, or hospital readmission within 30 days for the same diagnosis. Authors found no significant difference between groups when considering treatment failure. Authors concluded that uncomplicated diverticulitis can be successfully managed with a short course of intravenous followed by oral antibiotic therapy [40]. Lastly, given the publication date and underlying limitations for current use, the third trial reviewed by Shabanzadeh and Wille-Jorgenson will not be discussed in depth [41]. In summary, Shabanzadeh and Wille-Jorgenson concluded that no definite evidence exists for the use of antibiotics to treat CT-confirmed uncomplicated diverticulitis and that further studies on the topic are warranted to confirm these results [38].

De Korte and colleagues conducted a multi-center, retrospective, case-control study to assess the effect of antibiotics on failure rates of conservative management for mild colonic diverticulitis [42]. Conservative management was defined as restriction of oral intake, intravenous fluid rehydration and observation. Patients were assigned to one of two groups: those who received antibiotic therapy or those who received conservative management and no antibiotic therapy. Treatment failure was defined as the need for percutaneous drainage of abscess due to clinical deterioration and/or the need for urgent or emergency surgery. A total of 272 patients were included in the final analysis; 81 received antibiotic therapy and 191 received conservative management. Antibiotic regimens consisted of either a combination of piperacillin and metronidazole if admitted to a surgical ward or intravenous amoxicillin-clavulanic acid if admitted to a medical or gastrointestinal ward. Antibiotics were continued for a period of 7–10 days, depending on patient progression. The mean length of follow up was 50 months (range 12–100); during that time period, treatment failure rates did not differ significantly between groups, as 6% of patients randomized to antibiotic therapy and 4% of patients randomized to conservative management failed treatment. Although not significantly different, recurrence rates were higher in the antibiotic treatment group (15% *vs.* 7%; *p* = 0.055). Authors concluded that antibiotics may be omitted in select patients with mild colonic diverticulitis but may be beneficial when there is a marked inflammatory response or elevated temperature. Additionally, authors urge further trials to evaluate antibiotic therapy for mild diverticulitis while incorporating clinical and serologic findings [42].

### 3.5. Conclusion

A growing body of evidence suggests that antibiotic therapy in cases of CT-confirmed uncomplicated diverticulitis does not produce better outcomes than conservative medical management alone. Larger, randomized trials are needed to confirm these initial findings.

## 4. Cholecystitis

### 4.1. Epidemiology/Pathogenesis

Acute cholecystitis affects 2%–4% of the population and represents one of the most frequent hospital gastrointestinal admissions [43]. In 90% of cases, cholecystitis is the result of cystic duct obstruction by gallstones. Complete and persistent obstruction results in biliary stasis and inflammation which leads to gallbladder enlargement and wall thickening. Bacteria may infect the inflamed gallbladder and if left untreated, the gallbladder wall may become necrotic and gangrenous [44]. Risk factors for the development of acute cholecystitis include increasing age, female gender, obesity, rapid weight loss, diabetes and pregnancy [45]. Medications known to increase risk of gallbladder disease include progesterone, estrogens, fibrates, narcotics, anticholinergics, dapsone, ceftriaxone, erythromycin, and ampicillin [46]. Acalculous cholecystitis, which is an acute necroinflammatory disease of the gallbladder, occurs in 10% of cases and more commonly effects elderly males [47]. Risk factors for acalculous disease includes surgery, critical illness, ischemia, total parenteral therapy, AIDS and motility disorders [48].

### 4.2. Microbiology

Bile is typically sterile or contains a low inoculum of bacteria [44]. Bacteria can ascend from the duodenum into the biliary tract or by hematogenous spread from the portal vein. Bacteria is present in gallbladder bile in 35%–65% of patients with acute cholecystitis [49]. The presence of bacteria within gallbladder bile may be an indicator for more severe disease and a risk factor for infectious post-operative complications [50]. The most common bacteria isolated from bile cultures in community-acquired infections include *E. coli*, *Klebsiella* spp., *Enterobacter* spp., *Enterococcus* spp., and *Streptococcus* spp. Anaerobes are only isolated in 1% of cultures, however, emphysematous cholecystitis, identified when there is air in the gallbladder wall, is most commonly caused by gas-forming anaerobes [4,51]. Similar isolates are recovered from health care-associated infections with *E. coli* and *Klebsiella* spp. being most common. However, these infections tend to have a higher incidence of *Pseudomonas* spp. in addition to *Enterococcus* spp. An international study of 116 medical centers worldwide reviewed resistance patterns for patients with acute cholecystitis [52]. Of the population studied, 96.3% had community-acquired infections, with Gram negative organisms isolated in 70% of cases (*E. coli* 46.2%, *Enterobacter* spp. 9.2%, and *K. pneumoniae* 8.7%) and Gram positive bacteria in 24.3% (*Enterococcus faecalis* 34.4%, *Enterococcus faecium* 17.2%, *Streptococcus* spp. 15.6%), respectively. Surprisingly, 17 of the 21 total resistant isolates identified were from community-acquired infections, as ESBL *E. coli* or ESBL *Klebsiella* spp. accounted for 14 of 17 isolates. Multivariate analysis demonstrated health care-associated infection, inadequacy of empiric antibiotic therapy, and recent antibiotic use were significant risk factors for resistance. This highlights the importance of monitoring antimicrobial resistance patterns in both the inpatient and outpatient setting [52].

### 4.3. Clinical Presentation/Diagnosis

The main symptoms of acute cholecystitis are intermittent pain, tenderness and guarding in the right upper quadrant associated with possible nausea and vomiting. Murphy sign is present when a patient stops breathing due to pain while the gallbladder is being palpated during a deep inspiration. The diagnosis of acute cholecystitis requires local and systemic signs of inflammation and positive imaging studies. Systemic signs of inflammation include fever, leukocytosis (WBC > 10,000 cells/mm^3^), CRP > 3 mg/dL, and/or a mild increase in bilirubin or liver function tests. Right upper quadrant ultrasound detects the presence of stones in over 95% of cases. Overall, the combination of both local and systemic signs of inflammation in combination with positive imaging findings has sensitivity and specificity of 91.2% and 96.9%, respectively [53]. Classification of disease is based on patients’ signs and symptoms and risk factors for health care-associated pathogens. According to the TG13, Grade I (mild disease) is defined as acute cholecystitis in a healthy patient with no organ dysfunction but with mild inflammation of the gallbladder. Grade II (moderate disease) presents with a leukocytosis (WBC > 18,000 cells/mm^3^), palpable tender mass in right upper quadrant, duration of symptoms >72 h, or marked local inflammation. Grade III (severe disease) includes patients with signs and symptoms of organ system dysfunction [53]. SIS-IDSA guidelines for the treatment of complicated IAIs classify community-onset health care-associated disease in patients with one of the following risk factors: presence of an invasive device at time of admission, history of methicillin-resistant *Staphylococcus aureus* (MRSA) colonization or infection, or history of surgery, hospitalization, dialysis, or residence in a long term care facility in the previous 12 months from the date of the culture. Patients with hospital onset as defined as an infection that occurs >48 h after hospitalization are also at risk for more resistant pathogens [2].

TG13 guidelines recommend obtaining bile and blood culture in all cases of acute cholecystitis, with the exception of Grade I cases; this differs from SIS-IDSA recommendations [2,54]. The rationale for obtainment of these cultures stems from the fact that positive bile cultures have been correlated with progression of disease. The obtainment of these cultures may also help streamline therapy when broad spectrum antibiotics are started empirically. However, blood cultures do not always correlate with bile cultures. In a study conducted by Bang and colleagues, patients with acute calculous cholecystitis had positive blood or biliary cultures 50.4% (70/139) and 21.6% (30/139) of the time, respectively. Only 50% of patients had the same organism in both biliary and blood cultures [55].

### 4.4. Treatment

A literature search was conducted using PubMed and the search terms “acute cholecystitis”, “cholecystitis” and “acute drug therapy”. The search was limited to adults and studies from 2010 to present. For the treatment of acute cholecystitis and other IAIs, source control is critical. The initial assessment includes a decision to perform early surgery in the form of a laparoscopic or an open procedure. Early laparoscopic cholecystectomy is the preferred treatment in a majority of patients. Clinical trials have shown a decrease in hospital length of stay and total hospital costs with early surgery [56,57]. On the contrary, delaying surgery is necessary when there is a higher risk of biliary tract injury which may occur when surgery is performed on an inflamed gallbladder. In these patients, antibiotic therapy, intravenous fluids, and pain control is initiated.

With the lack of well-designed randomized trials to guide practice, practitioners look to published guidelines for direction. The SIS-IDSA, WSES and TG13 guidelines recommend empiric antimicrobial therapy based on disease severity and location of acquisition (community-acquired *vs.* health care-associated) [2,3,4,51]. Recommendations for the treatment of cholecystitis are listed in Table 1.

SIS-IDSA guidelines recommend anaerobic coverage only in patients with biliary-enteric anastomosis, such as hepaticojejunostomy or choledochoenterostomies, which are used to bypass the biliary tract for the management of biliary obstruction or leakage [2]. TG13 antibiotic recommendations are similar to SIS-IDSA. One modification is the use of ampicillin-sulbactam in combination with an aminoglycoside for Grade I community-acquired cholecystitis [4,51]. The SIS-IDSA guidelines removed ampicillin-sulbactam due to increasing resistance identified in *E. coli*, thus the empiric use of ampicillin-sulbactam is dependent on individual hospital antibiograms [2]. Both SIS-IDSA and TG13 guidelines recommend that fluoroquinolones should be used only if susceptibility of the cultured isolate is known or the patient has a severe allergy to beta-lactam antibiotics, as increasing resistance rates limit their utility [2,4,51].

**Table 1:** Antimicrobial treatment recommendations for cholecystitis in adults.

Indication	SIS-IDSA [2]	WSES [3]	TG13 [4]
Community-acquired	Acute cholecystitis, mild-to-moderate severity ▪ Cefazolin ▪ Cefuroxime ▪ Ceftriaxone	Biliary IAI, stable, non-critical patients with no risk factors for ESBL pathogens ^a^ ▪ Amoxicillin-clavulanic acid IV ▪ Ciprofloxacin plus metronidazole	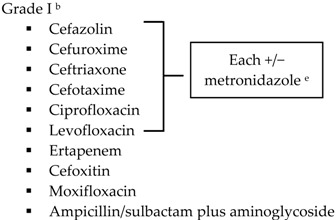
		Biliary IAI, stable, non-critical patients with risk factors for ESBL pathogens ^a^ ▪ Tigecycline	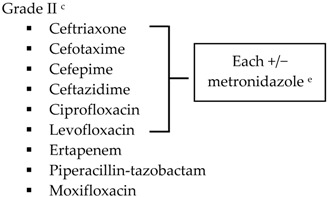
Community-acquired	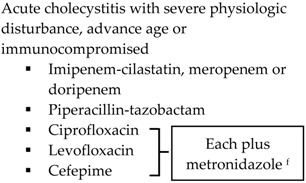	Biliary IAI, critically ill patients with no risk factors for ESBL pathogens ^a^ ▪ Piperacillin-tazobactam	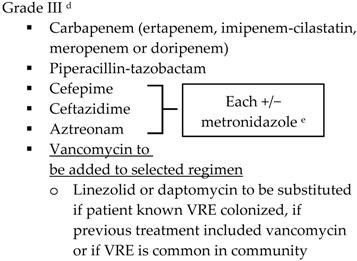
		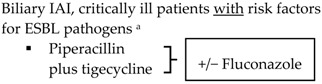	
Health-care associated	Biliary infection of any severity 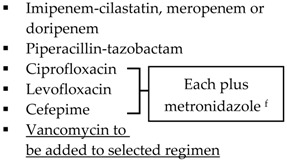		Health-care associated 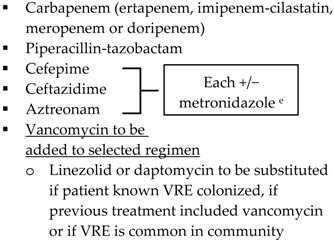
Other	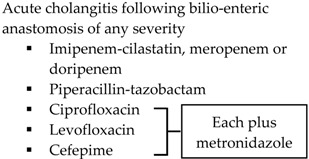		

SIS-IDSA: Surgical Infection Society and the Infectious Diseases Society of America; WSES: World Society of Emergency Surgery; TG13: 2013 Updated Tokyo Guidelines for the management of acute cholangitis and cholecystitis; ESBL: extended-spectrum beta-lactamase; VRE: vancomycin-resistant enterococci. ^a^ Risk factors for ESBL pathogens include prior exposure to antibiotics (especially third generation cephalosporins), serious comorbid conditions requiring concurrent antibiotic therapy, residence in long-term care facility, recent hospitalization, advanced age >65 years. ^b^ Grade I (mild disease): acute cholecystitis in a healthy patient with mild inflammation of the gallbladder but no organ dysfunction. ^c^ Grade II (moderate disease): presents with an elevated WBC (>18,000 cells/mm^3^), palpable tender mass in right upper quadrant, duration of symptoms >72 h, or marked local inflammation (including gangrenous cholecystitis, emphysematous cholecystitis, pericholecystic abscess, hepatic abscess or biliary peritonitis). ^d^ Grade III (severe disease): signs and symptoms of organ system dysfunction. ^e^ Anti-anaerobic therapy is warranted if a biliary-enteric anastomosis is present. ^f^ SIS-IDSA guidelines state “anaerobic therapy is not indicated unless a biliary-enteric anastamosis is present (B-II).”

In addition to the aforementioned stratification, WSES recommendations focus on risk factors for infections with ESBL organisms as there is an increase incidence of these pathogens in IAIs, even in the community setting [3]. Knowledge of the patients’ antibiotic history prior to admission and the rate of ESBL organisms in the community will help guide empiric therapy. For community-acquired infections in stable patients with ESBL risk factors, WSES recommends tigecycline over carbapenems to help decrease the risk of carbapenemase-producing Enterobacteriaceae [3]. In the United States, the use of tigecycline as a first line agent is limited due to the FDA black box warning of increased risk of mortality when tigecycline was used for both approved and unapproved indications [58].

### 4.5. Conclusion

As with other IAIs, source control is critical for the management of cholecystitis. Given the paucity of randomized trials to guide therapy, clinicians should utilize guideline focused recommendations as well as institution-specific antibiograms to develop empiric antibiotic regimens for the treatment of cholecystitis.

## 5. Antimicrobial Stewardship Initiatives

Antimicrobial stewardship programs (ASPs) can improve inappropriate antimicrobial use by ensuring appropriate empiric therapy, deescalating therapy, and decreasing inappropriate extended durations of therapy [6]. For all IAIs, risk factors for resistant pathogens, including but not limited to, high APACHE II score, health care-associated infection, prior antibiotic exposure and advanced age must be reviewed to help guide empiric therapy [59]. International guidelines were reviewed and a literature search was conducted using PubMed and a combination of the search terms “intra-abdominal infection”, “antimicrobial stewardship”, “antibiotic therapy” and “duration”. The search was limited to adults and studies from 2010 to present. Articles cited focused on antimicrobial stewardship initiatives for the treatment of IAIs. Additional publications were reviewed at the discretion of the authors.

Antimicrobial regimens recommended for appendiceal infections should be active against enteric Gram negative aerobic and facultative bacilli, Gram positive streptococci and obligate anaerobic bacilli. Treatment options for IAIs are listed in Table 2 and recommendations regarding the use of an antibiotics first strategy for the management of appendicitis are listed in Table 3 [2,3,20,60]. Reviewing published literature, SIS-IDSA guidelines recommend ertapenem as a treatment option for community-acquired mild-moderate IAIs, including perforated or abscessed appendicitis; however the Expert Panel expresses concern with the broad use of ertapenem, as it may accelerate the appearance of carbapenem-resistant Enterobacteriaceae, Pseudomonas and Acinetobacter species [2]. Additionally, secondary to a study conducted by Montravers and colleagues analyzing the microbiological and resistance profiles of IAIs, Leone and colleagues express concern regarding the overuse of anti-Pseudomonal carbapenems and the development of carbapenem resistance amongst Enterobacteriaceae [61]. To prevent the overuse of broad spectrum agents, ASPs must work with their microbiology department and utilize institution-specific antibiograms to develop guidelines for the empiric treatment of IAIs. Antibiogram focused guidelines will promote use of antimicrobials most likely to have adequate coverage for intra-abdominal pathogens. Dubrovskaya and colleagues developed a guideline for the empiric treatment of IAIs due to high rates of Enterobacteriaceae resistant to ciprofloxacin and ampicillin-sulbactam at their institution. Their primary endpoint was defined daily doses per 1000 patient days (DDD/1000PD) comparing pre- and post-guideline implementation periods. Authors found a significant decrease in intravenous ciprofloxacin use by 22.6 DDD/1000PD (*p* = 0.003) with no significant changes in ampicillin-sulbactam use (decrease by 11 DDD/1000PD; *p* = 0.8) nor piperacillin-tazobactam use (increase by 22 DDD/1000PD; *p* = 0.41). The hospital-acquired CDI rate, 30-day readmission rate and mean length of stay did not differ significantly between groups. Authors concluded that the new guideline showed an improvement in antimicrobial use with no significant changes in hospital metrics [62]. Meanwhile, Popovski and colleagues developed a multifaceted stewardship intervention that involved the development of IAI treatment guidelines based on local susceptibilities and an educational program that involved posters, pocket cards, and educational sessions for providers, nurses, and pharmacists [63]. This passive intervention significantly decreased the days of therapy per 1000 patient days (DOT/1000 PD) for ciprofloxacin and piperacillin-tazobactam. Comparing DOT/1000PD for pre- and post-intervention periods, ciprofloxacin use decreased from 221 to 74 (OR 0.3; 95% CI, 0.2–0.3; *p* < 0.001) and piperacillin-tazobactam use decreased from 116 to 67 (OR 0.6; 95% CI, 0.5–0.7; *p* < 0.001). However, ceftriaxone DOT/1000PD increased from 6 to 92 (OR 17; 95% CI, 10–25; *p* < 0.001). Although authors did not find a significant difference in duration of inpatient antimicrobial therapy, this stewardship initiative resulted in substantial change in prescribing practices that persisted after discontinuation of the intervention [63].

Acute appendicitis without evidence of perforation, abscess, or local peritonitis requires only prophylactic administration of antimicrobials for 24 h to prevent surgical site infections secondary to appendectomies. The 2013 Clinical Practice Guidelines for Antimicrobial Prophylaxis in Surgery provides guidance for antimicrobial selection and duration for use as prophylaxis before surgery [64]. Bowel flora constitute a major source for these organisms as *B. fragilis* and *E. coli* are the most frequent anaerobe and aerobe cultured, respectively. Recommendations for antimicrobial surgical prophylaxis secondary to uncomplicated appendicitis include cefoxitin, cefotetan, or cefazolin plus metronidazole. Routine use of broad spectrum agents like ertapenem, which is only approved by the FDA for prophylaxis of surgical site infections after elective colorectal procedures, is concerning due to the theoretical risk of promoting drug resistant organisms [64]. Lee and colleagues recently published results of a retrospective, case-control study of two surgical units with high rates of hospital-onset CDI. In this study, a total of 46 patients were diagnosed with CDI during the 15 month study period, and authors found that ertapenem prophylaxis was associated with a statistically significant increased risk of CDI [65].

There is an accumulating amount of evidence indicating that cases of community-acquired diverticulitis of mild-moderate severity can be managed without the use of antimicrobials [42]. Per guidance published by the American Gastroenterological Association Institute, in patients with CT-documented acute uncomplicated diverticulitis, antimicrobial therapy does not seem to improve symptoms or decrease the need for surgical intervention [66]. Additionally, antimicrobials may not decrease the development of complications nor curb recurrence rates [66]. ASPs can educate providers to consider withholding antimicrobial therapy and providing supportive care with close monitoring for patients with acute uncomplicated diverticulitis. Clinicians must assess each patient individually and ASPs should question the use of empiric antimicrobials when appropriate. When treatment is necessary, empiric antimicrobials should be tailored to disease severity and local resistance patterns and should include agents that have appropriate activity against *E. coli* and *B. fragilis*. Coverage for MRSA, *P. aeruginosa*, *Candida* spp. and resistant Gram negative bacteria are generally not needed and should be reserved for specific epidemiological patterns and patient risk factors [67]. The expanded Gram negative spectrum of broader, monotherapy antimicrobial agents, such as carbapenems and tigecycline, are often excessive in community-acquired diverticulitis of mild–moderate severity and their use may contribute to the emergence of antimicrobial resistance [2]. Based on local antibiograms, oral options for stable patients include a quinolone (either levofloxacin or ciprofloxacin) plus metronidazole, amoxicillin-clavulanic acid or trimethoprim-sulfamethoxazole plus metronidazole [40,41]. Community-acquired diverticulitis of high severity and health care-associated diverticulitis should be treated aggressively with antimicrobial regimens that have activity against multi-drug resistant Gram negative aerobic and facultative bacilli. Additional recommendations regarding antifungal therapy and empiric therapy targeting *Enterococcus* spp. and MRSA are provided in Table 2 [2,3].

**Table 2:** Antimicrobial treatment recommendations for appendicitis and extra-biliary IAIs in adults.

Guideline	Indication	Treatment
SIS-IDSA [2]	Community-acquired IAIs of mild-moderate severity including perforated or abscessed appendicitis	Single agent ▪ Cefoxitin ▪ Ertapenem ▪ Moxifloxacin ▪ Tigecycline ▪ Ticarcillin-clavulanic acid 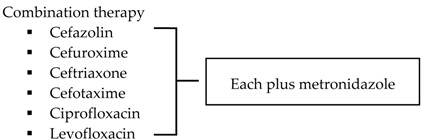
	Community-acquired IAIs of high risk or severity ^a^	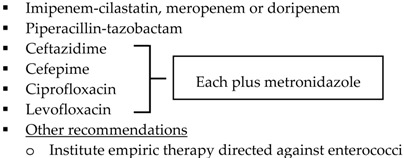
	Hospital-acquired IAIs	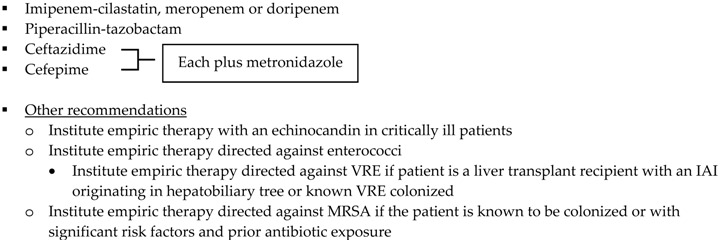
WSES [3]	Community-acquired extra-biliary IAIs	Stable, non-critical patients with no risk factors for ESBL pathogens ^b^ ▪ Amoxcillin-clavulanate IV ▪ Ciprofloxacin plus metronidazole
		Stable, non-critical patients with risk factors for ESBL pathogens ^b^ ▪ Ertapenem ▪ Tigecycline
		Critically ill patients with no risk factors for ESBL pathogens ^b^ ▪ Piperacillin-tazobactam
		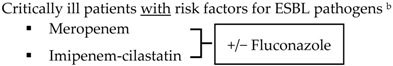
	Hospital-acquired extra-biliary IAIs	Stable, non-critical patients with risk factors for MDR pathogens ^c^ ▪ Piperacillin plus tigecycline plus fluconazole
		Critically ill patients with risk factors for MDR pathogens ^c^ ▪ Piperacillin plus tigecycline plus echinocandin ▪ [Imipenem-cilastatin, meropenem or doripenem] plus teicoplanin plus echinocandin

SIS-IDSA: Surgical Infection Society and the Infectious Diseases Society of America; WSES: World Society of Emergency Surgery; Echinocandins: anidulafungin, caspofungin or micafungin; VRE: vancomycin-resistant enterococci; MRSA: methicillin-resistant *Staphylococcus aureus*. ^a^ High risk or severity includes delay in initial intervention for >24 h, APACHE II score ≥15, advanced age, comorbidity and organ dysfunction, low albumin level, poor nutritional status, degree of peritoneal involvement or diffuse peritonitis, inability to achieve source control, presence of malignancy. ^b^ Risk factors for ESBL pathogens include prior exposure to antibiotics (especially third generation cephalosporins), serious comorbid conditions requiring concurrent antibiotic therapy, residence in a long-term care facility, recent hospitalization, advanced age >65 years. ^c^ Risk factors for MDR pathogens include nosocomial-acquired infections and prior exposure to antibiotics.

**Table 3:** Recommendations regarding the use of a nonoperative strategy in the management of appendicitis [20].

Guideline	Recommendation	Strength of Recommendation ^a^
SIS-IDSA [2]	▪ Nonoperative management may be considered for male patients, provided that the patient is admitted to the hospital for 48 h and shows persistent improvement in clinical symptoms and signs within 24 h while receiving antimicrobial therapy	A-II ^b^
	▪ Consider nonoperative management of patients with acute, nonperforated appendicitis if there is a marked improvement in the patient’s condition prior to operation	B-II ^c^
WSES [3]	▪ Appendectomies remain the treatment of choice for acute appendicitis ▪ For patients with uncomplicated acute appendicitis, antibiotic therapy is safe but less effective due to significant recurrence rates	1A ^d^
	▪ Evidence reveals that an interval appendectomy is not routinely necessary following initial nonoperative treatment of complicated appendicitis ▪ Interval appendectomies should be performed for patients with recurrent symptoms	2B ^e^
ACS [60]	▪ Surgery is the standard treatment for acute appendicitis ▪ Antibiotic treatment may be substituted for specific patients	-

SIS-IDSA: Surgical Infection Society and the Infectious Diseases Society of America; WSES: World Society of Emergency Surgery; ACS: American College of Surgeons. ^a^ Strength of recommendation differed between guidelines. ^b^ A-II: Good evidence to support a recommendation for use. ^c^ B-II: Moderate evidence to support a recommendation for use. ^d^ 1A: Strong recommendation, high-quality evidence. ^e^ 2B: Weak recommendation, moderate-quality evidence.

ASPs play an active role in de-escalating broad spectrum empiric antimicrobial therapy based on the results of intraoperative cultures. Consequently, narrowing antimicrobial therapy is often challenging without evidence of culture and susceptibility data, thus recommending empiric antimicrobial therapy based on location of acquisition and risk factors for resistant organisms are vital for ASPs. In hospital-acquired and health care-associated cases when anti-Pseudomonal and antifungal therapy is empirically initiated, de-escalation can occur when appropriately obtained cultures do not yield these pathogens. Although the obtainment of cultures for all appendectomy or cholecystectomy procedures is not recommended, cultures can be used to deescalate therapy as appropriate. In patients with cholecystostomy tubes, cultures should be obtained when inserted to help guide therapy and identify any new epidemiologic trends in pathogenic bacteria. Of importance, the SIS-IDSA guidelines do not recommend the addition of antimicrobials to cover culture results in patients with community-acquired IAIs who are responding to initial therapy [2]. ASPs can utilize this recommendation and educate health care providers to reconsider adding additional antimicrobial coverage in patients with improving hemodynamics.

Recommendations regarding duration of antimicrobial therapy for IAIs vary [2,3,4,51]. SIS-IDSA guidelines recommend 4–7 days of antimicrobial therapy for established IAIs, pending adequate source control [2]. For the treatment of diverticulitis, duration is dependent on the resolution of symptoms; guidelines suggest 7–10 days [40,41]. WSES guidelines reference that shorter antimicrobial durations have demonstrated similar cure and eradication rates when compared to longer regimens, as studies have shown a low likelihood of recurrence or failure when antimicrobials are withdrawn in patients who no longer show signs of infection [3]. TG13 guidelines suggest continuing antimicrobials for 24 h post-cholecystectomy in patients with Grade I infection and for 4–7 days for patients with Grade II or III infection, respectively. In patients with concomitant Gram positive bacteremia, TG13 recommends a minimum of 14 days of antimicrobial therapy [4,51]. For patients undergoing surgical procedures, intraoperative documentation and cultures of necrotic or inflamed tissue can help guide definitive antimicrobial selection and duration. ASPs should consult operative notes to help determine duration of therapy given achievement of source control and extent of the underlying infection. Duration of therapy for IAIs should be reviewed daily in conjunction with patient hemodynamics and laboratory and imaging findings to identify populations that may benefit from short courses of postoperative antimicrobial therapy [53].

Nonetheless, discontinuing antimicrobial therapy based on clinical parameters is not always performed, as it is common for practitioners to use a predefined length of therapy. An observational study conducted by Rodríguez-Sanjuán and colleagues analyzed the frequency of surgical site infections in Grade II patients post-cholecystectomy receiving guideline recommended antimicrobials for various durations. Group 1 (*n* = 45) received 0–4 days of treatment, group 2 (*n* = 76) received 5–7 days, and group 3 (*n* = 167) received >7 days. There was no significant difference in the incidence of surgical site infections amongst groups through 30 days post-surgery follow up [68]. Sawyer and colleagues randomized patients with IAIs and adequate source control to receive antimicrobials for two days after resolution of fever, leukocytosis and ileus (control) *vs.* a fixed duration of 4 ± 1 calendar day (experimental). The primary outcome was a composite of surgical site infection, recurrent IAI or death within 30 days after source control procedure. The primary endpoint occurred in 21.8% of patients in the experimental group *vs.* 22.3% of patients in the control group although the median duration of antimicrobial therapy was significantly shorter in the experimental group (4.0 days *vs.* 8.0 days; *p* < 0.001). While authors did not stratify outcomes based on origin of IAI, the appendix was responsible for 13.1% and 15.1% of infections in the control group and experimental group, respectively. Of note, target enrollment was not reached as an interim analysis showed nearly identical outcomes and continued funding was considered futile. Thus, although the trial did not meet power and proof of equivalence cannot be certain, the study provides promising evidence that a shorter, fixed course of antimicrobial therapy is similar to longer durations after resolution of signs and symptoms [69]. Catena and colleagues conducted a randomized controlled trial comparing three days of ertapenem *vs.* ampicillin-sulbactam in patients with community-acquired IAIs who received surgical intervention within 24 h of diagnosis [70]. Patients received one dose preoperatively and therapy continued postoperatively but was stopped on day three if the patient was afebrile, WBC normalized, and bowel sounds were present. Of the 142 patients enrolled, 54 patients presented with acute cholecystitis with phlegmonous, gangrenous, or perforation with a localized fluid collection. Treatment success was not stratified based on presenting diagnosis, but overall success occurred in 97% (69/71) of patients treated with ertapenem *vs.* 86% (61/71) of patients treated with ampicillin-sulbactam (*p* = 0.03). Additionally, more patients in the ampicillin-sulbactam group experienced superficial or deep postoperative surgical site infections (*p* = 0.03) [70].

## 6. Conclusions

Appendicitis, diverticulitis and cholecystitis are three types of IAIs that are commonly treated with antimicrobial agents. Broad spectrum empiric therapy and extended treatment courses can contribute to bacterial resistance and CDI. Patients suffering from IAIs should be managed in a manner respective of international guidelines [2,3,4,51]. Clinicians and health care providers should be aware of the treatment recommendations discussed by policy leaders to ensure positive patient outcomes. ASPs should utilize local antibiograms and epidemiology data to ensure proper empiric antimicrobial therapy for patients suffering from IAIs.
